# Assessment of Caspofungin use at a Tertiary Teaching Hospital and compliance with IDSA guidelines and FDA labelings

**DOI:** 10.1016/j.jsps.2021.12.005

**Published:** 2022-01-05

**Authors:** Abrar F. Alshehri, Thamer A. Almangour, Abdullah A. Alhifany, Abdulaziz Alhossan

**Affiliations:** a Department of Clinical Pharmacy, College of Pharmacy, Umm Al-Qura University, Makkah, Saudi Arabia; b Department of Clinical Pharmacy, College of Pharmacy, King Saud University, Riyadh, Saudi Arabia

**Keywords:** Caspofungin, Utilization, Evaluation, Use, Indication, Dose

## Abstract

**Objective:**

The aim of this study is to evaluate the utilization pattern of Caspofungin in an academic tertiary care hospital in Riyadh, Saudi Arabia.

**Methods:**

This is a retrospective study, conducted at King Saud University Medical City, Riyadh, Saudi Arabia. Adult patients who received Caspofungin from January 2015 to December 2018 were included. The appropriate use of Caspofungin was evaluated according to the international guidelines and approved recommendations. Caspofungin doses were assessed according to the FDA-approved loading and maintenance doses as well as dose-adjustment per hepatic function for cirrhotic patients and drug-drug interactions. Cultures and laboratory tests were used to evaluate the appropriate duration of Caspofungin therapy.

**Results:**

388 patients were included. Caspofungin was inappropriately used in 253 (64%) patients. This included 78 (20%) due to inappropriate indication, 165 (42%) due to wrong dosage, and 10 (2%) patients who had a wrong duration of therapy.

**Conclusion:**

The rate of inappropriate use of Caspofungin was high. Hence, developing antifungal stewardship and drug restriction program is highly recommended.

## 1 Introduction

Caspofungin is a member of a class of antifungals termed echinocandins which are approved by the US Food and Drug Administration (FDA) in adult and pediatric patients (3 months of age and older) as empiric therapy for presumed fungal infections in febrile neutropenia and for treatment of invasive aspergillosis (IA) in cases that are refractory or intolerant to azole antifungal agents (“Cancidas (Caspofungin Acetate) Injection,” 2001; Patterson et al., 2016), as well as for treatment of severe *Candida* (*C.*) infections including candidemia, intra-abdominal abscesses, peritonitis, pleural space infections, and esophageal candidiasis (“Cancidas (Caspofungin Acetate) Injection,” 2001; Pappas et al., 2016). In addition, Caspofungin has been used off-label to treat oropharyngeal candidiasis, candida osteomyelitis, septic arthritis, endocarditis, suppurative thrombophlebitis, and as empirical therapy in septic shock (Pappas et al., 2016).

The echinocandins are preferred over azole antifungal agents for suspected or confirmed fungal infections due to *C. glabrata* or *C. krusei* (Pappas et al., 2016). Moreover, it is *in vitro* active against *Aspergillus* (*A.) fumigatus*, *A. flavus*, *A. terreus*, *C. albicans*, *C. guilliermondii*, *C. parapsilosis* and *C. tropicalis* (“Cancidas (Caspofungin Acetate) Injection,” 2001; Pappas et al., 2016). However, Caspofungin is inactive against *Mucoromycetes*, *Cryptococcus*, and *Fusarium* species nor achieves therapeutic concentrations in ocular tissues, central nervous system (CNS), and urine which may lead to Caspofungin prescribing for inappropriate indications (Pappas et al., 2016). Moreover, multiple factors contribute to the improper dosing of Caspofungin, such as drug interactions, the need for a loading dose, and weight-based and hepatic dose adjustment (“Cancidas (Caspofungin Acetate) Injection,” 2001; “Cancidas (previously Caspofungin MSD),” 2009). Duration of therapy also varies based on the type of fungal pathogen, source of infection, culture result of sterile body sites, immune status, clinical response, resolution of imaging, and adequacy of source control (Patterson et al., 2016). These factors contribute to the inappropriate duration of therapy for Caspofungin.

There is a paucity of literature evaluating the use of Caspofungin in terms of indication, dose, and duration of therapy despite its high potential to be inappropriately prescribed (Abdel Fattah et al., 2015, Alonso et al., 2009, Fabien et al., 2014, Nivoix et al., 2012, Pavese et al., 2007, Raymond et al., 2009, Valerio et al., 2015, Valerio et al., 2014, Zilberberg et al., 2010). Therefore, this study aims to evaluate the use of Caspofungin at a tertiary-based teaching hospital and compliance with Infectious Diseases Society of America (IDSA) guidelines (Fisher, 2011, Freifeld et al., 2011, Pappas et al., 2016, Patterson et al., 2016), FDA labeling (“Cancidas (Caspofungin Acetate) Injection,” 2001) and European Medicines Agency (EMA) recommendations (Cancidas (previously Caspofungin MSD), 2009).

## 2 Methods

### 2.1 Study design and variables

Patients who received at least one dose of Caspofungin from January 2015 to December 2018 at King Saud University Medical City (KSUMC), Riyadh, Saudi Arabia, were identified from the medical records and their charts were retrospectively reviewed. Pregnant women and children were excluded from the study. The research was approved by the Institutional Review Board at KSUMC (E-19-3872). The primary objective was to assess Caspofungin use and compliance to international guidelines and approved recommendations (see A.1).

A data collection sheet was created to collect demographic information, medical and medication histories, lab tests, and microbiological cultures. The child-Pugh score was calculated for cirrhotic patients to determine dose adjustment based on the severity of chronic liver diseases.

Drug interactions and dose appropriateness were assessed using a comprehensive drug interaction checker and reliable databases (e.g., Lexi-comp and Micromedex).

### 2.2 Statistical analysis

Descriptive statistics were used to summarize the data. Variables were presented in numbers and percentages. Mean and standard deviation (SD) were used to describe age, height, and weight. Statistical analyses were performed using STATA 15.1 (StataCorp LP, College Station, Texas, USA).

## 3 Results

Three hundred and eighty-eight adult patients were identified. The mean age was 54 ± 18, and 51% were male. Around 133 (34%) patients weighed > 80 kg, among which 104 had BMI ≥ 30 kg/m^2^. The mean level of albumin was lower than the normal range (22.7 ± 6.7), while the mean level of total bilirubin and the mean level of prothrombin time (PT) were higher than the normal range (50.3, ± 124.9) and (19.4 ± 10.9), respectively. (Table 1) Caspofungin was initiated as empiric therapy for septic shock in 184 patients (48%) and documented fungal infections in 131 patients (34%) (Table 1).

**Table 1:** Summary of baseline demographics and patient characteristics.

Variable	Overall (n = 388)
Age (years), mean ± SD	54 ± 18
Gender: (male), n%	197 (51)
Weight (kg), mean ± SD	74.5 ± 27.9
Weight (kg) > 80 kg, n%	133 (34)
Weight (kg) ≤ 80 kg, n%	255 (66)
Height (cm), mean ± SD	158.7 ± 25
Body mass index (BMI) category	
Underweight (BMI < 18.5 kg/m^2^), n%	23 (6)
Normal (BMI 18.5 to 24.9 kg/m^2^), n%	121 (31)
Overweight (BMI 25 to 29.9 kg/m^2^), n%	107 (27.5)
Obese (BMI ≥ 30 kg/m^2^), n%	90 (23)
Obese (BMI ≥ 40 kg/m^2^), n%	41 (11)
Obese (BMI ≥ 50 kg/m^2^), n%	5 (1.5)
Lab results:	
Albumin (g/L), mean ± SD	22.7 (±6.7)
INR, mean ± SD	1.66 (±1.6)
PT (seconds above the normal range), mean ± SD	19.4 (±10.9)
Total bilirubin (mg/dl), mean ± SD	50.3 (±124.9)
Co– morbidity:	
Non– cirrhotic disease, n%	357 (92)
Cirrhotic disease, n%	31 (8)
Child-Pugh class A, n	0
Child-Pugh class B, n	10
Child-Pugh class C, n	21
Justification to initiate Caspofungin:	
Empirical therapy in FN, n%	33 (8)
Empirical use in septic shock, n%	184 (48)
Septic shock with risk factors, n	154
Septic shock without risk factors, n	15
Urosepsis, n	15
Fungal infections, n%	131 (34)
Aspergillosis infections, n	3
*Candida* infections, n	128
Candidemia, n	26
Intra-abdominal infection, n	84
*Candida* wound infection, n	5
*Candida* osteomyelitis, n	3
Candidiurea, n	2
Pneumonia, n	2
Endocarditis, n	2
Esophageal candidiasis, n	2
Others, n	2
No justified indication, n%	40 (10)

BMI: body mass index, INR: international normalized ratio, PT: prothrombin time, FN: Febrile neutropenia.

Cultures were positive in 50 patients (12.8%) and *Candida albicans* was the most frequently isolated pathogen. More details about the sources of infections and etiologic pathogens are listed in Table 2.

**Table 2:** Type of fungal pathogen and source of infection

Variable	No. of patients
*Candida albicans*	
Abdominal fluid, n	9
Blood, n	7
Sputum, n	2
Urine, n	1
Wound, n	3
*Candida glabrata*	
Abdominal fluid, n	3
Blood, n	10
Urine, n	1
Wound, n	2
*Candida krusei*	
Blood, n	3
*Candida parapsilosis*	
Blood, n	2
*Candida tropicalis*	
Blood, n	4
*Aspergillus* spp.	
Blood, n	2
Sputum, n	1

The overall inappropriate utilization was found in 253 (64%) patients, at which inappropriate indication, dose, and duration were found in 78 (20%), 165 (42%), and 10 (2%), respectively. More details about the reasons for inappropriate utilization for Caspofungin are listed in Table 3. Caspofungin use was considered appropriate in 21 patients with severe liver disease (Child- Pugh C), Caspofungin use was considered appropriate in 133 patients weighed > 80 kg.

**Table 3:** Reasons for inappropriate utilization of Caspofungin, N = 388.

Type of inappropriate utilization	Number of inappropriate utilizations	Number of patients died
**Inappropriate dose**	165	72
Missing maintenance dose	46	20
No loading	40	11
Child- Pugh B not adjusted to 35 mg daily	4	2
Drug interaction not adjusted to 70 mg daily	14	8
Inappropriately adjusted to 35 mg	59	31
Inappropriately adjusted to 70 mg	2	None
**Inappropriate indication**	78	26
Urosepsis	15	5
Unknown	40	12
Septic shock without risk	15	5
Candiduria	2	1
Aspergillosis without contraindication to other agents	1	1
Wound *Candida* with no contraindication to azole	4	3
Esophageal candidiasis with no de-escalation to azole.	1	None
**Inappropriate duration**		
Candidemia	10	3

The projected cost saving for 645 doses used in the inappropriate indication was 1,075,615.62 SAR (286,830.83 USD) (see A.2).

## 4 Discussion

To our knowledge, this is the first study to evaluate the utilization pattern of Caspofungin in terms of indication, dose, and duration. This study showed an overall inappropriate utilization of caspfungin and lack of compliance to international guidelines and approved recommendations at a tertiary teaching hospital, mainly, in terms of dosing and indication.

Several studies showed similar pattern of inappropriate utilization of antifungal agents, without exclusively investigating Caspofungin use (Abdel Fattah et al., 2015, Alonso et al., 2009, Fabien et al., 2014, Nivoix et al., 2012, Pavese et al., 2007, Raymond et al., 2009, Valerio et al., 2015, Valerio et al., 2014, Zilberberg et al., 2010). Nevertheless, the mean percentage of inappropriate use of Caspofungin was reported to be around 51%, which is almost comparable to our results (Abdel Fattah et al., 2015, Fabien et al., 2014, Nivoix et al., 2012, Pavese et al., 2007, Zilberberg et al., 2010).

Noteworthy, in our study, we had 21 patients with severe liver disease who received at least one dose of Caspofungin. Despite the absence of recommended dose for this population by the manufacturer, we categorized this dosing as appropriate according to a pharmacokinetic study which concluded that severe liver impairment does not necessitate further dose reduction compared to moderate liver impairment (Gustot et al., 2018).

Interestingly, few patients received Caspofungin with inappropriate dose adjustment due to the acute changes in liver parameters accompanied with their critical condition which later normalized after admission. Hence, the acuity of the patient status and the abnormality of lab parameters upon admission should be considered before dosing Caspofungin.

Moreover, 60% of the patients included in our study were categorized as overweight or obese who have received sub-therapeutic dosing of Caspfungin per the 2004 EMA recommendations (Cancidas (previously Caspofungin MSD), 2009). Nevertheless, this recommendation was based on two pharmacokinetic studies with small number of patients reported a decrease in serum trough concentrations (C_24_) in overweight individuals (Mistry et al., 2007, Stone et al., 2004). On the other hand, subsequent studies assessed Caspofungin concentrations in overweight individuals showed contradicting results (Hall et al., 2013, Muilwijk et al., 2014, Nguyen et al., 2007, Ryan et al., 2011, Würthwein et al., 2013).

Caspofungin was found to be used in 2 patients with candiduria, and 15 patients with urinary sepsis, in spite of the insufficient data to support the use of Caspofungin in *Candida* renal parenchymal infections, nor long-term follow-up to assess recurrence of *Candida* cystitis (Fisher, 2011).

Implementation of antifungal stewardship (AFS) program showed significant improvement in Caspofungin dosing; however, no significant improvement was reported in terms of indication and duration (Lachenmayr et al., 2019). Further, AFS programs showed improvment in antifungal use, cost saving and patients' survival (López-Medrano et al., 2013, Micallef et al., 2015, Ramos et al., 2015, Valerio et al., 2015). López-Medrano, et al showed that the dispensed defined daily doses (DDD) and expenditure of Caspofungin was reduced by 20% after implementing AFS program (López-Medrano et al., 2013). Additionally, the in-hospital mortality decreased after the implementation of AFS program compared with the previous period (López-Medrano et al., 2013, Ramos et al., 2015, Valerio et al., 2015).

Our study is not free from limitations such as being conducted retrospectively at a single tertiary care center, the difficulty in evaluating the duration of Caspofungin regimens, the de-escalation of Caspofungin to oral antifungal options, and the difficulty in assessing patients’ clinical improvements. Lastly, the causes behind Caspofungin misuse were not investigated.

## 5 Conclusion

As a conclusion, Caspofungin is highly likely to be inappropriately prescribed in terms of dosing and indication. Implementation of AFS program is of high necessity to improve Caspofungin utilization.

## Declaration of Competing Interest

The authors declare that they have no known competing financial interests or personal relationships that could have appeared to influence the work reported in this paper.

## Figures and Tables

**Table A.1:** Appropriate and inappropriate criteria for the use of Caspofungin.

	Appropriate use	Inappropriate use
Indication	● Empiric therapy in adult patients with persistent FN or recurrent fever ≥ 38 °C after 4–7 days of starting FN treatment with no identified source of fever (Freifeld et al., 2011)	● As single agent or as first choice in treating invasive aspergillosis (IA) without any contraindication to other antifungal agents
		● Reported Caspofungin hypersensitivity
	● First choice to treat *Candida* infections: (Pappas et al., 2016)	● Reported Caspofungin resistance (e.g., Caspofungin-resistant *C. parapsilosis*, or Caspofungin-resistant *C. glabrata*) (Pappas et al., 2016)
		● Candida urinary tract infections (unless complicated by disseminated candidiasis) (Freifeld et al., 2011, Kalra and Raizada, 2009)
	- Severe *Candida* infection (e.g., hemodynamically unstable or with organ involvement). Mild *Candida* infection, with recent azole exposure or contraindication	● Empirical use in septic shock (without the criteria mentioned in the appropriate use)

	I. Documented culture with *C. krusei*, or fluconazole-resistant *C. glabrata*	

	II. Prolonged QT or drug interaction (Patterson et al., 2016)	

	● Alternative choice for esophageal candidiasis and unable to tolerate oral agent (Pappas et al., 2016)	

	● Alternative choice (salvage therapy) to treat invasive aspergillosis (IA) in combination with other antifungal in patients who are refractory or intolerant of other antifungal agents (e.g., amphotericin B and azole) (Patterson et al., 2016)	

	● Empirical therapy in septic shock with risk factors for candidemia such as: (Pappas et al., 2016)	

	- Recent abdominal surgery	

	- Recent treatment with broad-spectrum antibiotics	

	- Immunocompromised (e.g., chronic corticosteroids or other immunosuppressants, neutropenia, malignancy, organ transplant, dialysis, and parenteral nutrition)	


Dosing	● Adult standard dose: 70 mg on day 1, then 50 mg once daily	● Missing loading dose
	● Moderate liver insufficiency (Child-Pugh class B): 70 mg on day 1, then 35 mg once daily	● Missing maintenance dose
	● Concurrent use with (rifampin, carbamazepine, dexamethasone, efavirenz, nevirapine, or phenytoin):70 mg once daily	● No dose adjustment performed per liver function or when interacting drug is given concomitantly
Duration	Note: Duration of Caspofungin treatment should be determined by clinical response, resolve imaging and the adequacy of source control	● Every other day culture was not taken after the last positive blood culture with candidemia infection
		● Appropriate duration criteria were not met
	- *Candida*: (Pappas et al., 2016)	

	I. Candidemia:	

	a. Continue for 14 days after the last negative culture	

	b. Consider de-escalation after 5–7 days for patients who are clinically stable and have negative repeated blood culture	

	II. Endocarditis: as initial treatment, followed by de-escalation (total duration ≥ 6 weeks)	

	III. Osteomyelitis & osteoarticular infections: 2 weeks, followed by de-escalation (total duration 6–12 months).	

	IV. Intra-abdominal: guided by clinical response and the adequacy of source control.	

	V. Esophageal candidiasis: 14–21 days, de-escalation if tolerate oral.	

	VI. Empirical: Caspofungin discontinued after 4–5 days of negative cultures or no clinical improving. Continue for 2 weeks in patients who improve.	

	- FN: with negative culture: until there is reversal of immunosuppression and clinical improvement	

	- Aspergillosis: normalization of antigenemia, resolution of clinical and radiological findings	

FN: febrile neutropenia; IA: invasive Aspergillosis.

**Table A.2:** Number of days and doses in inappropriate indication.

Inappropriate indication	
Total number of days of therapy (DOT) with inappropriate indication*	**661**
Overall number of doses in the inappropriate indication	**645**
Number of 70 mg dose used in the inappropriate indication	**114**
Number of 50 mg dose used in the inappropriate indication	**489**
Number of 35 mg dose used in the inappropriate indication	**42**

*including days of missing doses.

## Appendix A

See Tables A.1 and A.2.
